# Magnesium Sulfate Combined with Nifedipine Is Effective in Pregnancy-Induced Hypertension and Reduces Levels of Serum β2-Microglobulin and Retinol Binding Protein 4

**Published:** 2019-12

**Authors:** Jie SONG, Ruihong LAN, Humin GONG, Linmei ZHENG, Yang YANG, Dahua YI, Enze HU

**Affiliations:** 1. Department of Obstetrics, Hainan General Hospital, Haikou, P.R. China; 2. Department of Pharmacy, the Sixth Hospital of Wuhan, Wuhan, P.R. China

## Dear Editor-in-Chief

Pregnancy-induced hypertension (PIH) is a common disease in pregnant women that occurs mostly 20 weeks after pregnancy, and about 12% of maternal deaths are associated with PIH (1). If not properly treated, it not only damages organs of the pregnant woman, but also adversely affect the placenta of the fetus (2). At present, PIH is difficult to treat due to its unclear specific pathogenesis.

Clinically, magnesium sulfate is a common drug for PIH, which ameliorates the blood supply of organs, reduces blood pressure, expands blood vessels, as well as improves the circulation of the body (3). Nifedipine is effective in improving hypertension symptoms, inhibiting excitability of cardiomyocytes, protecting myocardial function, and controlling blood pressure (4). Labetalol and nifedipine have antihypertensive effects and play a role in controlling blood pressure in pregnancy patients with chronic hypertension (5). Oral nifedipine lowered blood pressure faster than intravenous labetalol during hypertensive emergencies (6).

In this study, a total of 204 PIH patients admitted to Hainan General Hospital, Haikou, China, were divided into two groups: Study group (magnesium sulfate combined with nifedipine, n=104), control group (magnesium sulfate, n=100). After treatment (Table 1), the study group showed significantly higher effective rate than the control group, which indicated that magnesium sulfate combined with nifedipine had better efficacy in treating PIH and alleviated the symptoms of patients.

**Table 1:** Comparison of effective rate, adverse reactions, and pregnancy outcome [n(%)]

***Efficacy***	***n***	***Markedly effective***	***Effective***	***Ineffective***	***Effective rate (%)***
Study group	104	69 (66.35)	25 (24.03)	10 (9.62)	90.38
Control group	100	46 (46.00)	34 (34.00)	20 (20.00)	80.00
χ^2^ value	-	-	-	-	4.383
*P* value	-	-	-	-	0.036
Adverse reactions	n	Ausea and vomiting	Cough	Facial flushing	Dry mouth
Study group	104	5 (4.81)	1 (0.96)	2 (1.92)	2 (1.92)
Control group	100	3 (3.00)	3 (3.00)	0 (0.00)	1 (1.00)
χ^2^ value	-	0.092	0.297	0.466	0.001
*P* value	-	0.761	0.586	0.495	0.973
Pregnancy outcome	n	Placental abruption	Premature delivery	Neonatal respiratory distress	Neonatal death
Study group	104	4 (3.85)	4 (3.85)	8 (7.69)	1 (0.96)
Control group	100	16 (16.00)	16 (16.00)	24 (24.00)	9 (9.00)
χ^2^ value	-	8.516	8.516	10.251	7.067
*P* value	-	0.004	0.004	0.001	0.008

There was no significant difference in the incidence of adverse reactions between the two groups, and the pregnancy outcome in the study group was better than that of the control group, suggesting that the combined use increased no adverse reactions and improved the pregnancy outcome of PIH patients, but the mechanism remained unknown.

PIH causes varying changes in kidney functions because kidney is an involved organ in early PIH. Serum β2-microglobulin (β2-MG), a biomarker reflecting renal diseases, is stable under normal conditions and is absorbed in proximal renal tubules (7). Retinol binding protein 4 (RBP4) is a small molecule protein in blood and urine, which increases when abnormal kidney function occurs (8).

**Fig. 1:**
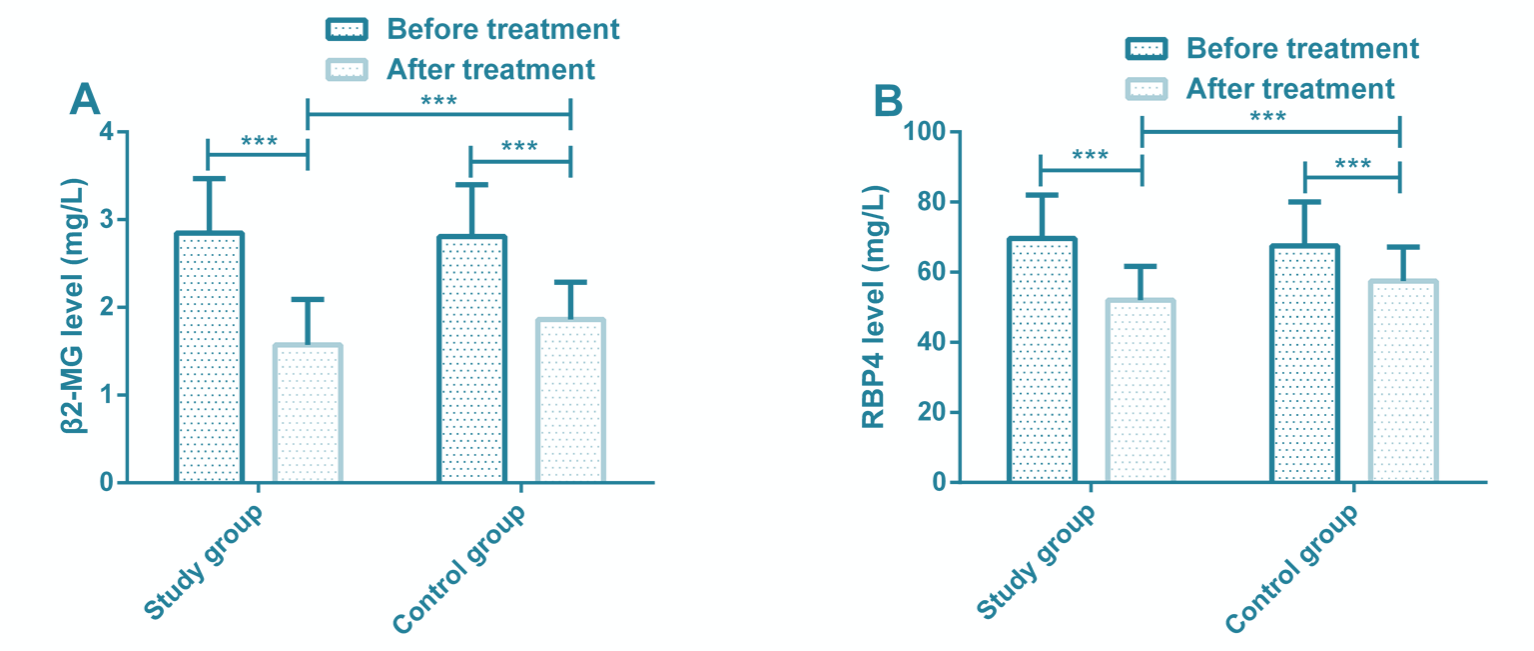
Comparison of serum β2-MG and RBP4 levels **(A)** Comparison of serum β2-MG level between the study group and the control group before and after treatment. **(B)** Comparison of serum RBP4 level between the study group and the control group before and after treatment. Note: ***P<0.001

In this study, the serum β2-MG and RBP4 levels in both groups decreased after treatment, and the decrease in the study group was more significant (Fig. 1). RBP4 level increased significantly in PIH patients, and the level in umbilical cord blood may be closely related to fetal growth (9).

Proteinuria component β2-MG in patients with preeclampsia and chronic kidney disease were significantly higher than those in healthy controls, and the level was positively correlated with 24h PRO (10). Thus, lowering β2-MG and RBP4 levels may be one of the therapeutic mechanisms of PIH. Yet, there are still deficiencies in our study. No in-depth discussion on the specific mechanism of β2-MG and RBP4 in PIH is conducted, which will be further addressed in future studies. Magnesium sulfate combined with nifedipine is effective in PIH, which down-regulates β2-MG and RBP4 levels and improves the pregnancy outcome of patients.
